# Evolving the theory and praxis of knowledge translation through social interaction: a social phenomenological study

**DOI:** 10.1186/1748-5908-4-26

**Published:** 2009-05-14

**Authors:** Carol L McWilliam, Anita Kothari, Catherine Ward-Griffin, Dorothy Forbes, Beverly Leipert, South West Community Care Access Centre Home Care Collaboration (SW-CCAC)

**Affiliations:** 1 School of Nursing, Health Sciences Addition, The University of Western Ontario, London, Ontario, N6A 5C1, Canada; 2 Faculty of Health Sciences, Arthur & Sonia Labatt Health Sciences Building, The University of Western Ontario, London, Ontario, N6A 5B9, Canada; 3 The South West Community Care Access Centre (SW-CCAC), 366 Oxford St W, London, Ontario, N7G 3C9, Canada

## Abstract

**Background:**

As an inherently human process fraught with subjectivity, dynamic interaction, and change, social interaction knowledge translation (KT) invites implementation scientists to explore what might be learned from adopting the academic tradition of social constructivism and an interpretive research approach. This paper presents phenomenological investigation of the second cycle of a participatory action KT intervention in the home care sector to answer the question: What is the nature of the process of implementing KT through social interaction?

**Methods:**

Social phenomenology was selected to capture how the social processes of the KT intervention were experienced, with the aim of representing these as typical socially-constituted patterns. Participants (n = 203), including service providers, case managers, administrators, and researchers organized into nine geographically-determined multi-disciplinary action groups, purposefully selected and audiotaped three meetings per group to capture their enactment of the KT process at early, middle, and end-of-cycle timeframes. Data, comprised of 36 hours of transcribed audiotapes augmented by researchers' field notes, were analyzed using social phenomenology strategies and authenticated through member checking and peer review.

**Results:**

Four patterns of social interaction representing organization, team, and individual interests were identified: overcoming barriers and optimizing facilitators; integrating 'science push' and 'demand pull' approaches within the social interaction process; synthesizing the research evidence with tacit professional craft and experiential knowledge; and integrating knowledge creation, transfer, and uptake throughout everyday work. Achieved through relational transformative leadership constituted simultaneously by both structure and agency, in keeping with social phenomenology analysis approaches, these four patterns are represented holistically in a typical construction, specifically, a participatory action KT (PAKT) model.

**Conclusion:**

Study findings suggest the relevance of principles and foci from the field of process evaluation related to intervention implementation, further illuminating KT as a structuration process facilitated by evolving transformative leadership in an active and integrated context. The model provides guidance for proactively constructing a 'fit' between content, context, and facilitation in the translation of evidence informing professional craft knowledge.

## Background

Gaps and delays inhibiting timely uptake of research for evidence-based health care continue to challenge implementation scientists. Accepting 'knowledge' as socially constructed [1] and 'evidence' as 'codified and non-codified sources of knowledge, including research evidence, clinical experience, ... professional craft knowledge, patient preferences and experiences, and local information' [2] complicates this task. These definitions lead implementation scientists to conceive of 'knowledge translation' (KT) as a dynamic process of exchange, synthesis, and ethically sound application of knowledge within a complex system of relationships among researchers and users [3].

This definition builds upon change theories [4,5], in particular, 'diffusion of innovation' [5], and numerous relevant theories from multiple disciplines [6]. From this perspective, KT is more than and different from 'science push', most frequently characterized as dissemination by researchers responsible and accountable for getting their scientific evidence to potential users. Likewise, this definition moves beyond the 'demand pull' approach, which emphasizes the initiative of policy, service delivery, and practice personnel in taking up and applying evidence, primarily through critical appraisal of research and/or continuing professional development. Rather, this definition suggests that KT is a social interaction process [7] between and among researchers and users, encompassing user participation [8], and considerations of the context, the evidence, and the facilitation process as essential components [2,9,10].

Despite the growing awareness of the complexities of the KT process [11,12], to date, implementation scientists have uncovered little knowledge about effective methods and approaches. While recent directions [2,8,10] have advanced KT theory and practice, largely, this literature reflects traditional post-positivist assumptions espousing discrete linear processes and reductionistic conclusions about cause and effect [2]. Considerations of the context, the nature of the knowledge in question, the process of KT, and the interaction of these three elements of KT endeavours seldom are emphasized [2]

Yet process evaluations of the implementation of complex interventions, or 'deliberately initiated attempts to introduce new, or modify existing, patterns of collective action in health care' [13] have recognized that programs are shaped by their human implementers, their vision of change, and the veracity of that vision. For example, implementation scientists [14] have developed realist evaluation, which focuses not on what works, but on what works for whom in what circumstances, and in what respects and how [14]. This strategy has been successfully used [15] to uncover social and other contextual impediments to and facilitators of successful implementation. Such work invites knowledge translators to adopt conceptualizations of knowledge, evidence, and KT as human processes fraught with all of the challenges of human subjectivity, dynamic interaction, and change within a complex context. Such conceptualizations are consistent with social constructivism, which views knowledge, and indeed, all human understanding, experience, and realities to be socially constructed through interactions amongst people [16].

In keeping with the assumptions and beliefs of social constructivism, we used a two-cycle participatory action approach for our KT intervention, intended to promote the uptake and application of tacit 'how to' knowledge. The evidence encompassed principles of an empowering partnering strategy for service delivery and care. In the first action cycle, we described the barriers and facilitators encountered [17]. In the second action cycle, our aim was to elicit greater depth of understanding of subjectively experienced social action, in this instance, the intricacies of participatory action KT. We selected social phenomenology as a methodology that directs attention specifically toward understanding how things are ordinarily experienced with the aim of representing these experiences as typical socially-constituted patterns [18,19]. The purpose of this paper is to present the findings from the latter cycle, the holistic interpretation of which constitutes a theoretical model affording new insights into the theory and practice of social interaction KT.

In the accountability-oriented context of health care, hierarchical, authoritative, and power-laden relationships within health services organizations foster the inclination to 'push' evidence to practice. Such push, however, is met with professional relationships and boundaries when those down the line have experiential or tacit knowledge that might conflict with the evidence being pushed [20,21]. As these opposing contextual forces may stifle KT, there is increasing recognition that successful KT requires a work context that affords those inclined to push and those involved in 'pull' an opportunity to together engage in critical reflection, shared decision-making [22-25], and collective construction of the best processes toward envisioned outcomes.

The research evidence that constitutes the content of KT endeavours further challenges KT in the health sector [2,10]. While much research evidence is factual and technical in nature, a large portion of it, particularly from qualitative investigation, relates to refining professional craftsmanship, that is, the tacit, 'how to' knowledge and humanistic understanding that constitutes the art of practice [26,27]. Increasingly, too, such craftsmanship is expected of multiple diverse disciplines who share responsibility and accountability for care. To complicate matters further, professionals inevitably combine or replace research results, predominantly syntheses of randomized controlled trials, with tacit or 'how to' knowledge and humanistic understanding acquired from experiential learning, professional training and socialization, information about the local context [10,28,29], procedure manuals [30], and/or colleagues [29,31]. Practitioners' professional esteem comes from this professional knowledge base and its application [32]. Furthermore, notions of 'scope of practice' and uni-disciplinary social and cognitive boundaries [33] may lead to the prioritization of discipline-specific knowledge. Hence, new evidence, especially evidence related to tacit knowledge that has relevance across disciplines, may challenge practitioners' self-esteem and openness to trans-disciplinary evidence, in general impeding the translation of practice-related research evidence [17,21].

Two contemporary frameworks currently inform KT in such circumstances. The first, Promoting Action on Research Implementation in Health Services (PARiHS) [2,9,10], suggests three essential considerations: the evidence, the context, and facilitation. The evidence is described as encompassing research findings, clinical experience, and professional craft knowledge (that is, tacit 'how to' knowledge). The context ideally reflects sympathetic values and beliefs, openness to change, strong leadership, decentralized decision-making, role clarity, and appropriate monitoring and feedback. Facilitation by skilled external and internal personnel is recommended to enable teams and individuals undertaking KT to analyze, reflect upon, and change their own attitudes and behaviours, and particularize research findings [2].

The PARiHS framework identifies a set of variables and relationships that merit consideration in implementing KT, and in conducting diagnostic and evaluative measurement of such endeavours [2]. However, the PARiHS framework neither factors in the individual attributes of those expected to use the research evidence, nor provides guidance about how to address these very real human elements throughout the KT process.

In a second approach, the Knowledge to Action (KTA) framework, Graham and colleagues elaborate two KT process components: knowledge creation and knowledge application [8]. Knowledge creation is described as the tailoring of research-based knowledge through synthesis or aggregation of this evidence, and, subsequently, the creation of tools for clear, concise user-friendly presentation formats designed to influence what potential users do with the evidence. As such, this component of the KTA framework constitutes 'science push' [7]. Knowledge application, the KT intervention, is described as an action cycle consisting of deliberately-engineered dynamic phases. Organizational groups identify problems and issues, search for relevant research, and critically appraise this evidence to determine its validity and usefulness to address the problem at hand. These groups customize the selected research evidence to their particular situation, assess the barriers to its use, then select, tailor, and implement interventions to make change, and monitor and evaluate the outcomes achieved. Knowledge uptake and application are sustained through a feedback loop, accommodating local and external knowledge. As such, this component is in keeping with the 'demand pull' perspective [7].

The KTA framework [8] accommodates different types of knowledge, but affords limited insight into how one might combine the 'what' of KT (that is, evidence, context, and facilitation, as elaborated by the PARiHS model) with the 'how' (that is, the participatory action cycle) of KT. Graham *et al. *suggest that the KT process is complex and dynamic and that the two KTA components have blurred, permeable boundaries. However, within the knowledge creation component, the push described overlooks the well-known vagaries of human nature and behaviour of users in reaction to such push [21]. Contextual considerations, too, are objectively handled, through *a priori *conscious adaptation and tailoring of the knowledge to the local context, with due consideration of contextual barriers. The multi-layered (macro-, meso- and micro-) dynamic nature of context, and its potential as an active ingredient of the KT process are overlooked. The fallibility contained within the expectation that users will willingly adopt the role of pulling the process of knowledge application forward and avoid getting caught up in power relationships is not contemplated.

Process evaluations of new policy initiatives and complex intervention implementation suggest important considerations. For example, a process evaluation of the introduction of the expert patient programme in the National Health Service in the United Kingdom [15] identified the need to attend to action at different levels of the organization, interaction between key agencies and personnel, and ongoing effort to evolve strategies that work in an ever-changing context. A naturalistic study of the implementation of best practice guidelines across 11 health care organizations [34] uncovered the importance of both mobilizing the professional workforce to actively implement and monitor the implementation of guidelines, and providing leadership support for an evidence-based practice culture. Another investigation of the same complex intervention implementation identified the importance of group interaction, champions, teamwork and collaboration, as well as inter-organizational collaboration and networks to facilitate guideline implementation [35].

Investigation of participatory action research (PAR) also has uncovered insights of relevance to social interaction KT. PAR has been found to integrate KT with the innovation development and adoption process. Specifically, the PAR process enables participants to take an innovation and adapt it to their context, to engage in critical reflection to achieve this adaptation, and to work behind the scenes to encourage involvement and commitment [36], thus empowering participants through an iterative, locally responsive process of devolved responsibility. However, the researchers also observed challenges, including diverse perspectives, concerns, and unequal power relationships both amongst individual participants and in the context outside of the organization.

Investigation of the spread of innovations premised on health care research similarly has exposed challenges potentially relevant in undertaking social interaction KT. In two comprehensive qualitative case studies, Ferlie, Fitzgerald, Wood, and Hawkins found that the social and cognitive boundaries between health professions impeded spread, as individual professionals tended to operate within their own disciplinary paradigms and communities of practice [33]. Resistance to uptake was particularly marked where professional roles and identities were strong, social distances between disciplines were great, and research traditions, conceptions, agendas, and questions were markedly different. This finding cautions against undertaking KT within heterogeneous provider groups.

While these findings are informative, investigation specifically focused on social interaction KT approaches has been limited. Through participatory observation of 30 large, multi-year projects featuring either community-university alliances for health research (n = 19) or interdisciplinary health research teamwork (n = 11), Birdsell, Atkinson-Grosjean, and Landry found that the approaches to KT emphasized exchange rather than synthesis or direct application of knowledge [37]. Contextual factors, including space and time issues, organizational impediments, and structural barriers affected the management of KT. Challenges to KT implementation included inadequate time, money, and effort. Predictors of KT success included: adequate budgets and resources; researchers' early engagement with potential 'users'; pre-existing relationships; shared governance; previous KT activity; role clarity; team communication; and mechanisms for peer connection, relational learning, and the co-creation of knowledge. The researchers concluded that formal partnership agreements, early engagement of potential 'users', and consideration of researcher rewards and recognition would facilitate KT.

Pilot testing of our initial application of a participatory social interaction approach to KT uncovered many of the same barriers and facilitators. Findings suggested the need for ongoing attention to macro (organizational), meso (team), and micro (individual) barriers and facilitators to KT. Mobilizing the organization's fiscal and human resources for KT, team-oriented trust, support, relationships, work and ownership, and individuals' attitudes, motivation, time for and sustained commitment to KT proved challenging [17]. Participants recommended that project leaders create more opportunities for relationship-building and group discussions across all components of the organization, as well as enhanced communication channels and mechanisms.

Overall, research to date suggests several important considerations to guide the development of social interaction approaches to KT. However, there is little direct evidence to inform implementation scientists about the process of going about achieving this aim. This paper begins to address this gap, specifically answering the research question: What is the nature of the process of implementing KT through social interaction?

## Methods

### Design

The KT intervention, the social phenomenon under investigation, was premised on the principles of participatory action. To explore the nature of participants' enactment of this KT process, we used social phenomenology [18,38]. Social phenomenology is undertaken to overcome naïve acceptance of the social world and its idealizations and formalizations as ready-made and meaningful beyond all question. Social phenomenology treats thought and action as intersubjective, integral parts of human existence, behavior, symbols, signs, social groups, institutions, and legal and economic systems, all embedded in history, time, and space [18,38]. Thus, social phenomenology is both consistent with the belief that reality is socially constructed and appropriate for the exploration of participatory action [19].

### The context

The project was undertaken collaboratively with six home care programs in the process of government-mandated amalgamation into one organization [17] that employed a total of 1,470 FTE providers (200 case managers, 390 nurses, 840 personal support workers, 35 therapists, 5 social workers) to serve approximately 16,000 clients across a 22,000 square kilometer urban/rural area within south western Ontario, Canada. With extensive role overlap, the multiplicity of providers normally worked in isolation despite their shared involvement and espousal of a team approach to care. The amalgamated organization had adopted a mission, philosophy, strategic plan, and service delivery model informed by the research evidence that constituted the content of this KT initiative.

### The evidence

The evidence from 18 years of collaborative applied research with these and other agencies [39-46] informed practice principles for fostering empowering partnering with clients and care team members. The principles promoted consciously attending to building relationships, being client-centered, using critical reflection, engaging and building on one another's strengths, and fostering clients' and team members' contributions of personal knowledge, skill, and decision-making ability as partners in service delivery and care. Hence, the evidence constituted tacit practice knowledge that necessitated shifting from an expert approach to providing treatment and care for medical problems to one enabling health as a resource for everyday life, by building on strengths and broadening the focus beyond physical status. As might be anticipated in the context of the western scientific world, where professionals have knowledge and roles that define their identities [47] and status [32], investigation had already demonstrated that the intended evidence-based practice refinement might invoke resistance to KT [21].

### KT intervention

The KT intervention was designed as a participatory action approach [48-52]. Participants were engaged in: critically reflecting on the research evidence and its implications for practice; identifying opportunities for change; using the evidence and personal knowledge of their work and context to formulate strategies for change; implementing and evaluating changes; and acting to institutionalize and diffuse these changes [50], consistent with the training and reinvention thought to be essential to adoption of innovation [53].

The nature of and fit between the study context and the research evidence [2], as well as existing KT frameworks [8,10,54], theory [55-61], and evidence [62-65], were important considerations in contextualizing and planning the KT intervention. Specifically, the PARiHS framework guided our assessment of the context and evidence, and informed our decision to involve both internal and external facilitators.

As the evidence was related to tacit practice knowledge foundational to all health practitioners' roles, we recognized that uptake might also be promoted experientially through the KT process. In addition to the publications, audiovisual presentations, illustrative case studies, and consultations provided in the first action cycle [17], in this second cycle, the researchers (who had functioned as external facilitators in the first action cycle) served as resource personnel and provided backstaging [66]. The latter included a binder containing draft agendas, critical reflection facilitation guides, and group process evaluation forms, as well as consultations to groups and their facilitators, and mentoring in the critical reflection process.

Despite previous research suggesting that uni-professional groups might be more conducive to KT [33], the action groups were intentionally heterogeneous in composition. Trans-disciplinarity is increasingly deemed important in contemporary knowledge production [67-69], where the knowledge to be co-constructed is intended to be applied in interdisciplinary service delivery and care.

Action groups set their own meeting times at approximately monthly intervals over an eight-month period. Draft agendas were adapted to incorporate their KT efforts into their everyday work. Meetings were facilitated by group-selected members, who used the facilitation guide. Without exception, managerial members were chosen for this role, which was designed to foster critical reflection on the practical integration of the research evidence and real-life service delivery. All action groups involved other organizational members, as appropriate, to develop, implement, and/or test their selected action strategies. Action groups were networked through a leadership implementation committee comprised of group-selected representatives and facilitators. Through monthly meetings and a one-day evaluation workshop, this committee facilitated and integrated knowledge exchange, uptake, spread, and application across the organization, its action groups, and individual members.

### Research methods

Investigation of this KT initiative was approved by the Research Ethics Board of the University of Western Ontario.

### Sample

The nine geographically-constructed multi-disciplinary action groups who participated in the second cycle of the KT process constituted the convenience sample for this study. The sample thus was comprised of the 203 home care program personnel, including a mix of providers (35 nurses, 14 therapists, 50 personal support workers, 2 social workers), decision makers (75 case managers, 15 supervisors, 3 administrators), and research resource persons (9, one per action group).

### Data collection

Over the eight-month, second-cycle KT intervention, each of the nine action groups was asked to audio-tape three meetings of their choice, one to reflect their KT process at the outset of this cycle (meetings one, two, or three), one in the middle of the cycle (meetings four, five, six, or seven) and one at the cycle's end (meetings eight or nine). This purposeful sampling strategy was designed to promote participants' involvement in capturing their enactment of the KT process across the cycle. As meetings varied in length both within and across groups (range, one to two hours; mean, one hour, 36 minutes) a total of 36 hours of audio-taped data was obtained for transcription. Researchers made supplementary informal field notes of participatory observations of meeting contexts, group dynamics, or other details of nuances and subtleties that might facilitate interpretive analysis of the audio-taped transcriptions.

### Data analysis

All transcribed data were entered into N-Vivo for qualitative data management. In interpretive analysis, researchers immerse themselves in the data and try to make sense of what is going on, iteratively reviewing, and re-reviewing data for themes and/or patterns, and ultimately crystallizing a holistic interpretation [70-72]. In social phenomenology, interpretive analysis calls for identification of first-level constructs reflecting common-sense experience of the intersubjective world in daily life [38], then second-level objective ideal-typical constructs, or distanced, disinterested-observer interpretations of the 'subjective meaning of the actions of human beings from which the social reality originates' [38]. Findings therefore constitute a non-generalizable 'typical construction' [38], comprised of the subjective experience of the participants and the intersubjective interpretations researchers make of that experience.

Individual and team effort included analysis of the transcribed data to identify first-level constructs capturing participants' intersubjective experience of KT, specifically the four patterns identified as findings. Field notes associated with the corresponding transcripts were used to assist in crystallizing the interpretations of these first level constructs. Interpretive analysis then proceeded to a second-level typical construction of the meaning of the actions of this social phenomenon, specifically the PAKT model [38].

### Authenticity

The principal investigator kept a record of ideas generated in analysis sessions for the purpose of facilitating the team's on-going iterative, interpretive process. Once a preliminary analysis was achieved, the researchers presented this to the leadership implementation committee, including representatives of the action groups, a practice called 'member checking'[73], and to other researchers and collaborators not directly involved in the action groups, a process called 'peer review'[73]. These techniques afforded feedback to help ensure that findings captured the lived experience authentically and made sense to others [73].

## Results

The findings of this interpretive investigation revealed participants' experiences of the intersubjective process of KT, thereby informing a typical construction of the KT process, in accordance with the methodology of social phenomenology [67,68]. KT was both contextually embedded and socially constructed over time through four patterns of enactment, as portrayed in the following sub-sections.

### Overcoming barriers and optimizing facilitators

Participatory interaction amongst diverse group members in the study optimized participants' mutual efforts toward confronting the barriers they attributed as impeding efforts toward empowering partnering with clients. As well, this interaction enabled the participants to socially construct facilitators, transcending competing perspectives and potential conflict between and amongst people representing macro-, meso- and micro-components of the organization. Throughout their KT process, participants collectively constructed an organization encompassing their co-created, shared beliefs and assumptions about their organizational identity, one that increasingly espoused the principles of empowering partnering. These findings are congruent with previous theoretical work linking social interaction to organizational evolution through identity construction [74,75] and research describing participants' social construction of barriers in implementing organizational change [76]. The following data illustrate this social construction:

Facilitator: ... We [action group participants] had a little discussion about ... how the first person in [provider in the client's home] needs more time than we often allot for that first visit [participants' social construction of macro/organizational barrier to KT], so if we really want to put forward client-driven care, we really need to back it up with authorized time so that they [individuals at the front line] can [provide it] ...[participants' social construction of a macro/organizational facilitator to KT]

Front-line provider: ... Our senior director [provider agency representative] talked to ... _______ [senior manager of provider agency contracts], who deals with all of the provider agencies ... to manage all the contracts. [meso/team level social construction of a facilitator to KT] ... there was some enthusiasm from him. ... We said, 'Could we have an hour [for the first visit]? [micro/individual social interaction in effort to facilitate KT] ... She said 'No.' [socially constructed organizational barrier by giving voice to a competing perspective]

Facilitator: I guess ... it's probably up to you folks to kind of make some recommendations about how the implementation should be rolled out. [facilitator invites team-level social construction of facilitation to overcome potential conflict].

### Integrating science push and demand pull

Integrating both science push and demand pull also occurred within the process of social interaction, a pattern illustrated by data from another action group:

Facilitator [managerial]: ... [At] the last meeting ... we [managerial facilitator using the KT facilitation guide] asked you some specific questions to try and make sure we were covering different areas [*i.e.*, the evidence-based principles in the initial draft of case conferencing guidelines], so if you don't mind, I'm going to give you ... five minutes to read through those two pages and do some thinking yourself [to see] if there's anything that's a disconnect, or really sparks a creative thought ... for the development ... of [evidence-based]guidelines ... for [case] conferenc [ing]. [Science push on behalf of the organization]

Front-line participant [a practitioner, following critical reflection]: It's around the team or ... designating someone ... Just the word 'designate' sounds a little controlling. I wondered about 'seek someone willing to take notes', versus 'you are taking notes.' [Demand pull, requesting that the evidence-based knowledge inform the proposed practical application]

Front-line participant: ... It [the case conferencing protocol] would have to be restructured because ... the way we're doing it now ... is that you have the input of each person and ... the issues identified ... [in] kind of a synopsis ... and then the end result, ... and what the decisions were and what the plan to go forward is ... I think ... we're all adults, ... so if I can look at it [the detailed case conferencing notes] and have a copy and, you know, hash that ... over in my brain, ... then I can go back to it, and read it again, ... and then say ... 'I can do this' [decide an appropriate partnering strategy in accordance with the evidence-based principles] ... [Demand pull, a practitioner suggesting that the evidence-based knowledge be applied to refine the practice approach]

Front-line participant: I'm wondering if on the first bullet we could just add the words 'and shared' to make sure the client and family expectations are clear and that they're shared. [Demand pull]

Front-line participant: I had another thought ... [on] ensuring follow-up to the conference. There's something about supporting relationships and communication between providers to make sure that the conference result happens ... It's that whole ... enhancement of the relationship amongst the team. [Demand pull]

Facilitator: How do you do that? [managerial facilitator promoting demand pull]

Front-line participant: ... I don't know how we do it, but we can't just kind of come together at one time and then assume that we're all going to go our separate ways and ... do our part ... It's that whole fostering of communications and ... relationships between the providers involved ... and there's an encouraged piece and there's an allowed piece [a practitioner openly confronting science push] and I think that we do need to kind of table it as a discussion because, you know, ... you can't plan together and just expect it's going to happen without at least chatting about it now and again, or being able to chat about it. [front-line practitioner facilitates demand pull amongst action group participants]

Participants' effort to transcend science push and demand pull through social interaction was further revealed by open discussion in another action group, as follows:

Front-line participant: ... You can't just come in and impose a structure [i.e., client-driven care approach to case conferencing] on an area and then tell other people that they're supposed to follow what you say when you've never done their job yourself [opposition to science push] ... I think that it's so important that we have everybody who's doing the job together, because you need to get the information from the people on the ground ... If you don't have everyone's input, you know, you could impose something that just isn't going to work. [voicing belief in and expectation for demand pull]

### Synthesizing the research evidence with tacit and experiential knowledge

Participants' social construction of mutually-shared knowledge revealed a pattern of synthesis of their tacit professional craft knowledge, affective stances, experiential knowledge, practice strategies, and corporate memory of organizational structures, policies, and procedures, with the research evidence. One action group's construction of synthesized knowledge portrayed this pattern:

Facilitator: ... The original champions from phase one [of the KT project] ..., their method was a team case conference held ... in the client's home, and during that conference, the four principles of empowering partnering in the home were followed, ... those principles we just looked at. ... The results from the phase one group were that the client's quality of life improved as well as the client's and caregiver's coping abilities. They got together and they all talked about it, and they were able to come up with a plan of action that would work better for this client. [recollection of relevant experiential knowledge of pilot testing the evidence-based principles] ... In light of ... their ... experience and recommendations ..., we need to consider the pros and cons of each of the ... components [guidelines] that we have developed ... from their recommendations. [synthesis of experiential knowledge into the co-creation of a refined evidence-based direction] ... So, ... if we had a conference [using the guidelines] and we've worked it through, it's resolved ... If it's not resolved, the people working in policy and procedure [preparation] need to look at that. [promoting synthesis of the evidence-based direction with existing organizational policy]

Participant: But that would be up to a case manager more than likely. We wouldn't necessarily have input to that policy. [practitioner critically reflecting on the uptake of the proposed evidence-based direction, given experiential knowledge of standard operating practices]

Facilitator: I think ... [that] ... there's some judgement here ... I think we need to keep that in mind ...

Participant: ... I think that the whole thing is that anybody can call a ... case conference, even the client [facilitator and participant both integrating knowledge of the evidence-based principles to promote a synthesis with experiential knowledge, and ultimately, evidence-based refinement of case conferencing practices].

### Integrating knowledge creation, transfer and uptake throughout everyday work

As action group discussions unfolded, participants moved more naturally between knowledge creation, transfer, uptake, and application, addressing and integrating each component into everyday work, if and as appropriate, in no particular order. The following group discussion reveals this pattern within the KT process:

Facilitator [managerial]: So, when you go back to your team meetings or your agency meetings, would you feel comfortable talking about client-driven care and the partnering. ... Is there a plan that you can do that?

Front-line participant: We've already started. [Evidence-based knowledge transfer/dissemination beyond action groups] ... In a couple of our meetings, it's been brought up ... And we are working on some of the issues. [knowledge co-creation, drawing upon experiential knowledge from individuals across the wider organization for consideration along with the research evidence]

Participant Facilitator [managerial]: [We checked] to see what the ... policy was around [case] conferencing and there wasn't a lot there. It has some steps about how you call a conference, and what you record, and this sort of stuff, but it didn't have ... guidelines about what a conference should look like, ... that kind of stuff ... There wasn't anything to prevent us from being as creative as we wanted, whether its in the MIS [Ministry Information System], ministry definitions or within ... [organizational] guidelines. We could really do what we think makes sense [proceeding to contemplate knowledge application] as long as we can come up with a good plan that gets support from all of our agencies. [integrating knowledge creation, uptake and everyday work practices]

Thus, KT became a non-boundaried part of everyday work; neither KT nor any of its components had an identifiable beginning, ending, or place in a fixed sequence. Rather, participants pursued their everyday work, integrating their KT effort.

### The overarching construction of social interaction KT

**Figure 1:**
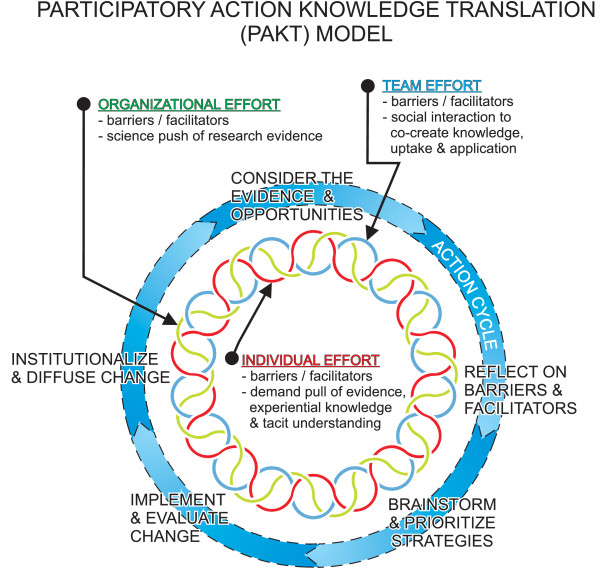
**Participatory action knowledge translation (PAKT) model**. Reprinted with permission, Journal of Change Management (2008), 8(34), 238.

Figure 1 depicts participants' holistic experience of the dynamically evolving KT process as a participatory action knowledge translation (PAKT) model, described in detail elsewhere [17]. Loosely following the action cycle, through the four social interaction patterns described in this paper, participants intersubjectively enacted a relational transformative leadership constituted simultaneously by both structure and agency, in keeping with structuration theory [77-79]. Structuration theory posits that the human agency of individuals who comprise an organization and the structure in which they operate are simultaneously constituted within a complex relational process in which neither has primacy. Structure is not outside of human agency, but exists only because of human agency, encapsulated in the PAKT model as organizational, individual, and team effort. Societal, system, and institutional directions, 'rules' and/or norms that govern individuals' communication and actions both shape and are shaped by individuals, who actively maintain and reproduce structure within society, systems, and institutions, a process called structuration.

Within this structuration process, the uptake and application of knowledge occur unconsciously, through taken-for-granted tacitly-enacted practices that become routinized and familiar, and most intentionally, by conscious evolution through social interaction focused on the co-creation of discursive knowledge. This third type of knowledge, over which individuals are assumed to exert control, was the focus of the PAKT process. This process of mutually engaging, shared enactment of transformative leadership enabled participants representing all components of the organization to more directly confront traditional boundaries and silos, barriers and facilitators, science push and demand pull to enact shared responsibility and accountability for promoting KT throughout everyday work. As previously described, this action reflected organization identity construction [74-76], in this instance, toward interpreting the principles of empowering partnering in everyday service delivery and care.

## Discussion

Interpretive research elicits insights from in-depth observation of real-life experiences. In this instance, study findings illuminate key features of an ideal typical construction of social interaction KT given the research content, context, and people involved. Firm conclusions about specific strategies and solutions for KT cannot be drawn. Indeed, the human nature of social interaction KT precludes straightforward replicable explanations of how to go about this process, which inevitably contains as many socio-political challenges as opportunities for success. Implementation science will therefore perhaps forever be as much art as science.

Nevertheless, the overarching experience of the intersubjective process of KT identified in this investigation, and the four patterns of structuration within it, may have applicability in the proactive design and implementation of KT of any evidence that informs the refinement of professional craft knowledge. In particular, study findings illustrate the importance of integrating the 'how to' with the 'what' of KT, that is, its content, context, and facilitation.

These findings enhance knowledge in the field of implementation science, particularly highlighting the relevance of principles that direct attention to social constructions as critical components requiring evaluation in the implementation of complex interventions [13,14]. Process evaluators suggest that careful consideration must be given to what the content may mean for those expected to accept and apply it, its implications for their goals, knowledge, self-confidence, relationships, responsibilities and accountabilities, their tasks, resources, rewards, and performance. As well, they emphasize the importance of context, and the fit of the content with this context, with due attention to practicalities, such as the resources, costs, and risks associated with uptake of the content in question, as well as organizational factors that may impact upon outcomes [13]. Additionally, process evaluation scientists direct extensive attention to group processes in organizational contexts, suggesting that attention to facilitation of group effort also may promote outcome attainment. These foci parallel those identified in the PARiHS framework, underscoring their relevance in illuminating the process of social interaction KT, as discussed in the following subsections.

### The content

The content of this KT process constituted professional craft knowledge on 'how to' work with clients using evidence-based principles of empowering partnering. The KT approach was intentionally designed as a direct application of these principles, in particular setting a stage on which participants could exercise agency and professional judgement in integrating these principles into everyday work. As portrayed by study findings, this approach afforded participants the opportunity to be empowered, to exercise 'responsible agency in the production of knowledge', thereby reducing their 'risk of co-optation and exploitation .... in the realization of the plans of others' [80]. The KT action groups also enacted within-group partnering and iterative, contextually and situationally sensitive responsiveness [36] in their effort to implement the empowering partnering principles in everyday service delivery and care.

Thus, the KT process in and of itself constituted experiential learning of the evidence related to the professional craft knowledge of empowering partnering. As the 'how to' of practice is not simply a matter of cognitive uptake and application of facts, but rather, is learned through situated discerning action encompassing interpretation, formation, contextualization, and performance [26], this insight may have applicability for the translation of any evidence that relates to the craft of professional practice. For example, evidence regarding how to provide psychosocial support for clients who are suffering, how to function as an interdisciplinary team, or how to listen actively may be applied in creating a KT process design that similarly affords experiential learning of that content.

### The context

Many of the ideal contextual elements for KT identified by Kitson *et al. *[2] were apparent in the organizational context in which this study was undertaken. Organizational leaders not only were committed to the values and beliefs underpinning the empowering partnering approach and the KT process, but also had formally set the stage for organizational change to enact the evidence-based principles. Nevertheless, this work context contained many impediments to both the KT process and the uptake and application of the evidence [17] that had to be overcome.

As revealed in all four patterns within the KT process, consistent with the findings of another study [76], these barriers were overcome when participants enacted a more level playing field and transformative leadership. Throughout their social interactions, they openly and intentionally confronted organizational, team, and individual barriers, resolved conflict, mutually constructed facilitators and strategies, and transcended science push and demand pull. Generally, this social action allowed all who comprised the organization, and, hence the organization itself, a voice in co-constructing both the knowledge to be translated and approaches for translating it. Overall, participants and their agency rendered the context more compatible with the content and successful pursuit of KT.

This insight merits consideration in undertaking social interaction KT. The ideal context for KT may not exist in the real world of health care. Several studies have identified numerous factors which may either impede or facilitate KT, including attitudes and beliefs, time, resources and support, organizational structures and processes, leadership, roles, and interaction patterns [10,31,33,35,81-85]. To the extent that barriers and facilitators are social constructions, and hence, specific to people within their own context, intentionally engaging participants in creating a more ideal context may help to overcome 'real-world' limitations. Thus, a better context for KT may be achieved if participants are organized to enact a level playing field and enabled to inform one another about the challenges throughout the organization. This may help them to mutually confront barriers and optimize facilitators, and to integrate real and perceived responsibilities and accountabilities for science push and demand pull through social interaction. With this staging, as KT participants attend to and apply the KT content, the everyday organizational operating culture, hence, the organizational context for KT, may be socially constructed into one which has greater 'fit' [10] with the KT content, through what constitutes an on-going process of organizational culture change [17].

### The facilitation

Kitson *et al. *recommend facilitation of the KT process by skilled personnel both external and internal to the organization [2]. In this project, facilitation transpired more successfully through evolving the collective transformative leadership agency of the KT participants than through sole enactment of a formalized facilitation role.

As previously described, in this second action cycle, facilitation of the KT process initially was vested in a group-selected member. As it happened, all groups chose someone known to all as having a managerial position in the organization. However, as the KT process transpired, over time, all participants became more engaged in ways reflective of transformative leadership effort. Transformative leadership evolved more slowly, and perhaps less consciously, than did the refinement of the KT context through participants' agency. Nevertheless, to varying degrees at any one point in time and with different action groups, this notion of leadership gradually became the facilitation mode.

McPherson, Popp, and Lindstrom suggest that transformative leadership is difficult to achieve in the public service sector – the dual hierarchies of the organization and the professions within it make it difficult for individuals to move beyond traditional organizational thinking, policies, and management techniques [86]. In the first action cycle [17], the researchers had assumed the formalized role of external facilitator. But this approach seemed to reify mutually exclusive roles for the researchers as 'knowledge brokers' and the participants as 'knowledge users', sometimes creating we/they relationships. Having participants in this second action cycle together choose an internal facilitator offset this problem somewhat, rendering researchers more 'equal' group members. However, the majority of action group members were frontline practitioners accustomed to the formalized leadership of managers in their more hierarchical work context. Hence, the groups selected managers as the internal facilitators.

As revealed in the data presented herein, internal facilitators' effort to create a level playing field and to actively engage participants in the KT process helped to construct a context that enabled all to more comfortably contribute to the facilitation of KT. Over time, participants' enactment of transformative leadership roles evolved. Consistent with previously identified facilitation strategies [10], data capture facilitators' efforts to enable the contributions of participants by building from individuals' ideas, nurturing critical reflection, promoting mutual effort toward reconciliation and negotiation when barriers appeared, and by consciously attending to building relationships among all involved. The critical reflection facilitation guides and draft agendas provided by the researchers served as a template to guide this evolution.

Facilitation through developing and evolving transformative leadership enabled KT participants to create the ideal blend of KT content and contextual elements, synthesizing research evidence with their tacit professional craft and experiential knowledge, and adapting and integrating knowledge creation, uptake, and application into their everyday work. Having groups identify and enact their own KT action priorities undoubtedly enhanced the achievement of this aim, reflecting the intertwining of structure and agency. Undoubtedly, however, overcoming entrenched patterns and beliefs reflecting leadership and followership in accordance with positions in a traditional organizational hierarchy takes time, sustained effort, and patience.

## Conclusion

The findings of this study illuminate the relevance of structuration theory to social interaction KT. The PAKT model constructed through this interpretive investigation is premised on the academic tradition of social constructivism. The four patterns of the KT process uncovered through this investigation represent the praxis of structuration theory. Applying structuration theory to the theoretical understanding of KT afforded by the PARiSH framework adds 'how to' to the 'what' of KT theory and praxis. The PAKT model encapsulates a more sophisticated, active, and integrated notion of context [54] and a shared enactment of facilitation through transformative leadership. Its explication provides guidance for proactively addressing the content, context, and facilitation of the translation of professional craft knowledge, with due attention to constructing 'fit' between these components in the design and implementation of KT. The model also adds to the Graham *et al. *framework, exposing the essentiality of having both researchers and 'users' and all levels of the health care hierarchy together [8].

Much more qualitative and quantitative investigation is required to more definitively inform the theory and practice of KT. Many issues remain unresolved. Having participants rather than researchers tailor the evidence, the context, the process, and the facilitation of KT through structuration means uptake of modified research findings. Sharing responsibility and accountability for the KT process means shared responsibility and accountability for outcomes. Such sharing is equally challenging to achievement-oriented researchers and organizational decision-makers committed to promoting evidence-based practice, and to practitioners pursuing what they know intuitively and tacitly to constitute quality health care.

This challenge largely arises from questions of whether and why researchers and decision-makers should think that they have greater capability for responsibly and accountably achieving evidence-based outcomes than frontline practitioners, and whether, why, and how their control over the outcomes of frontline practitioners' efforts could and would make a significant positive difference. Several authors have both argued [10] and demonstrated that effective professional practice encompasses a melding of several types of evidence, including research, clinical experience, patient experience, and information from the local context [10,29,87], as well as patient preferences and professional values and beliefs [87,88]. Furthermore, in general, research to date continues to demonstrate that even when researchers and/or decision makers do assume full responsibility and accountability for ensuring the uptake and application of research evidence in more linear models of knowledge transfer, outcomes fall short of those intended [34,89,90]. To fully address concerns regarding accountability and responsibility, further research might compare outcomes of models such as PAKT with those of more traditional KT approaches.

Another unresolved issue is how patients/clients and their family caregivers, also key stakeholders in any health services KT [91-93], might be engaged. Perhaps most importantly, with due regard for structuration theory, application of the PAKT model in and of itself may be viewed as 'top-down' push, and/or a conformation to existing practice norms. This reality merits conscious attention in any effort to adapt or adopt this approach to KT.

Thus, the findings of this study do not afford a straightforward prescribed solution to KT. Nevertheless, insights regarding the applicability of structuration theory and the patterns of structuration that constituted the PAKT process may serve as a guide in executing the art of implementation science, with careful adaptation to the content, context, and people involved.

## Competing interests

The authors declare that they have no competing interests.

## Authors' contributions

CMcW led the project implementation, the interpretive analysis of the findings, and drafted the manuscript. AK refined intellectual content related to existing KT frameworks. CWG drafted and refined intellectual content related to structuration theory. All authors participated in the project implementation activities, data collection, and peer review and refinement of interpretive findings. All authors also contributed to draft refinements, and read and approved the final manuscript.
